# Cutaneous protothecosis in a solid organ transplanted patient^☆^

**DOI:** 10.1016/j.abd.2022.10.008

**Published:** 2023-04-27

**Authors:** Anama Di Prinzio, Marina Ruf, Ana C. Torre, Sofía V. Duran Daza, Victoria I. Volonteri, Viviana Flores, Luis D. Mazzuoccuolo

**Affiliations:** Italian Hospital of Buenos Aires, CABA, Argentina

Dear Editor,

A 35-year-old woman, with a history of cretinism and chronic kidney disease secondary to congenital urological pathology, received a kidney transplant at the age of 30. At 5 years, due to graft rejection, she required a new transplant and was started on prednisone 20 mg/day, tacrolimus 16 mg/day, and mycophenolate mofetil 250 mg every 8 hours.

During her admission for bacteremia secondary to urinary tract infection, an erythematous plaque with diffuse borders and discrete asymptomatic superficial scaling was observed on the anterior and lateral sides of her left leg (Figure 1, Figure 2).

**Figure 1:**
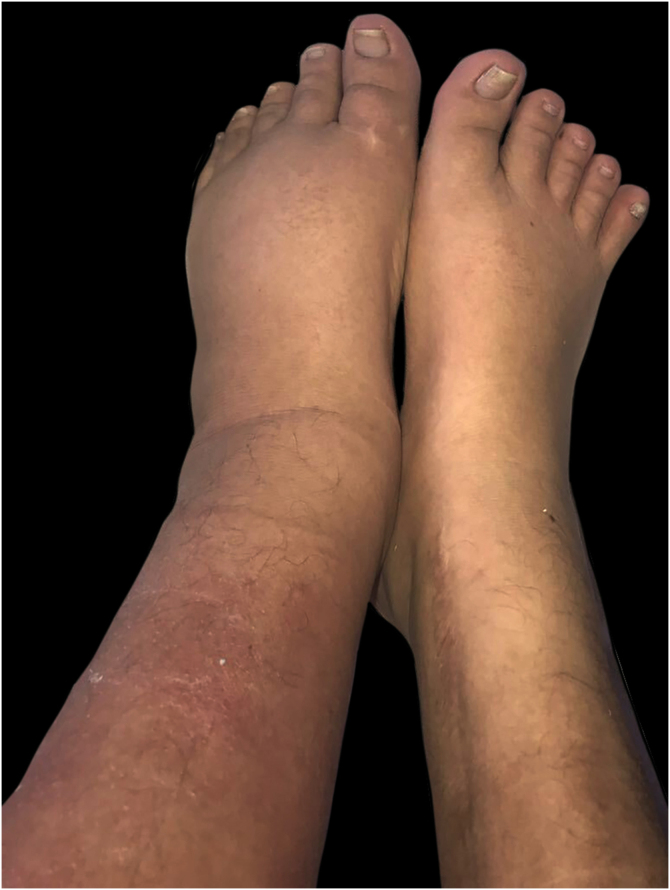
Asymmetry of the lower limbs due to an increase in the circumference of the left leg associated with erythema on its anterior region

**Figure 2:**
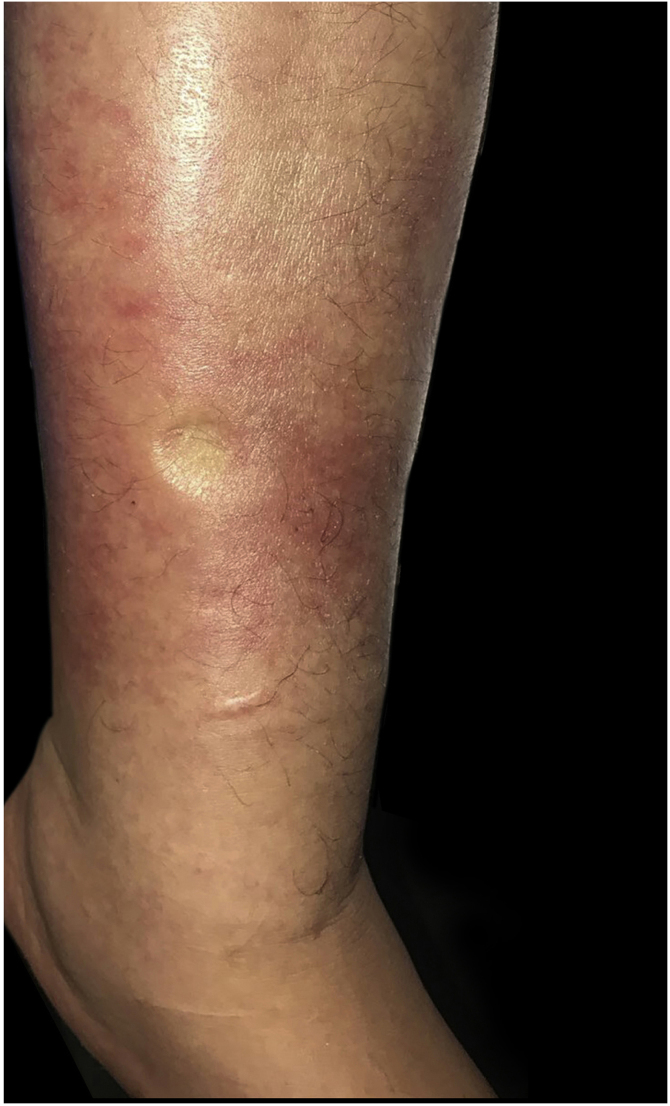
Erythematous and edematous plaque with diffuse borders on left leg. Edema with positive Godet's sign

Laboratory investigations showed leukocytes of 4900 mm^3^ with a relative neutrophilia of 94%. Arterial and venous Doppler ultrasound of the left lower limb showed lymphedema and increased echogenicity of the subcutaneous tissue. PET-CT revealed an increase in the density of the subcutaneous cellular tissue, accompanied by an inflammatory process. Skin biopsy was performed for histopathology and culture of bacteria, mycobacteria, and fungi. The first presented large, rounded structures, with multiple cytoplasmic septa, some with a morula-like appearance, and numerous sporangia with internal septa forming endospores (Figure 3, Figure 4). In culture, creamy white colonies were observed, some rough with depressed centers, compatible with *Prototheca spp*. (Fig. 5).

**Figure 3:**
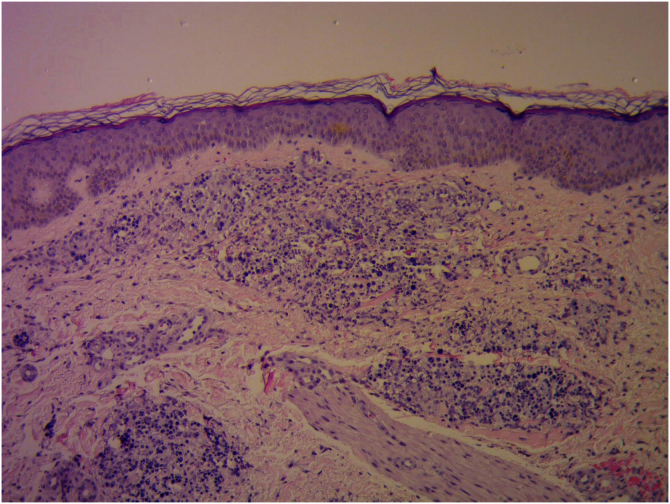
Granulomatous dermal infiltrate consisting of lymphocytes, histiocytes, multinucleated giant cells, and rounded structures, isolated or grouped, of different sizes, inside histiocytes and giant cells (Hematoxylin & eosin, ×100)

**Figure 4:**
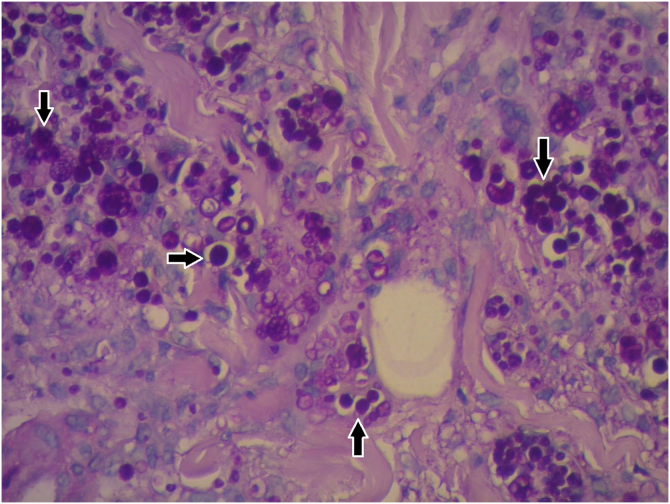
Histopathological characteristics (Hematoxylin & eosin, ×400): Rounded structures larger than the surrounding cells and multiple cytoplasmic septa, which gives them a morula-like appearance. Sporangia highlighted

**Figure 5:**
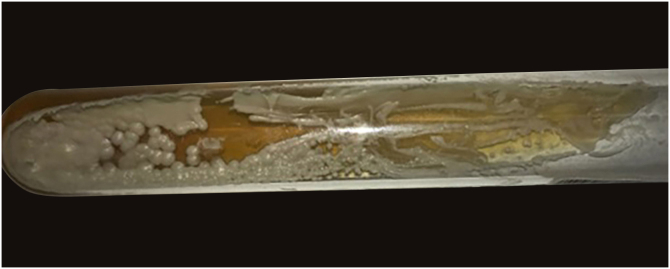
In culture, creamy white colonies were observed, some rough with depressed centers, compatible with *Prototheca* spp

Systemic treatment with liposomal Amphotericin B 200 mg/day was started, with an improvement of the skin lesions. Fourteen days later, he has switched to itraconazole 200 mg every 8 hours for 3 days and then continued at 200 mg every 12 hours. It evolved in a torpid way with partial improvement and intermittent worsening. At 9 months, a new skin culture was performed where the sensitivity of Prototheca was verified, and treatment was changed to fluconazole 200 mg every 12 hours and minocycline 100 mg every 12 hours.

Ultimately, she developed septic shock related to a urinary tract infection caused by carbapenemase-producing Klebsiella pneumonia. Antibiotic treatment was administered, but despite this, the patient developed multi-organ failure and died.

Protothecosis is a rare infection caused by algae of the species *Prototheca spp*. Within the species of the Prototheca genus, *Prototheca wickerhamii* and *zopfii* are the ones that most frequently affect immunocompromised hosts.^1^, ^2^

Prototheca infection ranges from indolent and localized skin involvement, soft tissue infection, olecranon bursitis in immunocompetent patients, to devastating disseminated infection with algemia and visceral infiltration with high mortality in immunocompromised hosts due to transplant, diabetes, HIV, and hematologic diseases.^2^, ^3^ The cutaneous form represents the most frequent manifestation (3 out of 4 patients). The lesions usually appear in areas exposed to traumatic implantation. It usually presents with poorly defined erythematous plaques, although less frequently it can manifest with nodular, pustular, warty, and ulcerated lesions.

Diagnosis is made by clinical suspicion, detection of characteristic structures in skin cultures, and microscopic examination. The definitive diagnosis of infection is usually based on the morphological identification of the organisms in culture preparations in wet slides and/or direct identification in tissue samples as in the reported case.^1^, ^2^, ^3^, ^4^

Its prognosis is good in almost 70% of cases. On the other hand, when it presents in a disseminated form, it has a worse prognosis, with high mortality.^1^, ^5^ The most commonly used medications are antifungals, including amphotericin B and systemic azoles. Amphotericin B is currently the first-line treatment in disseminated cases and in patients with severe underlying diseases or immunosuppression.^6^, ^7^, ^8^, ^9^

In conclusion, protothecosis is an infrequent infection with nonspecific skin manifestations, so in the presence of plaques, nodules, ulcerated or warty lesions in immunosuppressed patients, a skin biopsy should be performed for culture and histopathology to detect infectious agents. The initiation of adequate treatment prevents the progression of the disease.^5^, ^6^, ^7^, ^8^, ^9^, ^10^

## Financial support

None declared.

## Authors' contributions

Anama Di Prinzio: The study concept and design; writing of the manuscript.

Marina Ruf: The study concept and design; intellectual participation in the propaedeutic and/or therapeutic conduct of the studied cases.

Ana C. Torre: Data collection, or analysis and interpretation of data.

Sofía V. Duran Daza: Writing of the manuscript or critical review of important intellectual content.

Victoria I. Volonteri: Data collection.

Viviana Flores: Data collection.

Luis D. Mazzuoccuolo: Final approval of the final version of the manuscript.

## Conflicts of interest

None declared.
